# Impact of anemia on contrast-induced nephropathy (CIN) in patients undergoing percutaneous coronary interventions

**DOI:** 10.1007/s11255-012-0340-8

**Published:** 2012-12-07

**Authors:** Wen-hua Li, Dong-ye Li, Fei Han, Tong-da Xu, Yang-bing Zhang, Hong Zhu

**Affiliations:** Department of Cardiology, Affiliated Hospital of Xuzhou Medical College, No. 99 Huihai west Road, Xuzhou, 221002 China

**Keywords:** Anemia, Angioplasty, Contrast, Nephropathy

## Abstract

**Background:**

The aim of the present study was to assess the influence of anemia on the risk of developing contrast-induced nephropathy after percutaneous coronary angioplasty.

**Methods:**

Serum creatinine values were measured before and within 48 h after the administration of contrast agents. Contrast-induced nephropathy (CIN) was defined as an increase of ≥0.5 mg/dl or ≥25 % in serum creatinine concentration over baseline within 48 h after administration. Anemia was defined as hemoglobin <120 g/l in women and <130 g/l in men.

**Results:**

Among the 1,026 patients studied, 32 (3.1 %) developed CIN after procedure. CIN occurred in 6.3 % of the anemic patients and in 2.2 % of the non-anemic patients (*P* < 0.01). The incidence of CIN increased with decreasing of baseline estimated glomerular filtration rate (eGFR) in both the anemia and non-anemia groups. In patients with baseline eGFR <30 ml/min, a high proportion of both anemic and non-anemic patients experienced CIN (24.6 vs. 17.5 %). When baseline eGFR was 30–59 ml/min, the incidence of CIN in anemic patients was twofold higher than in non-anemic patients (7.9 vs. 3.8 %; *P* < 0.05). Multivariate logistic regression analysis found that baseline eGFR and baseline hemoglobin were independent predictors of CIN.

**Conclusion:**

Anemia is associated with a higher incidence of CIN in patients with moderate renal dysfunction. Patients with both preexisting renal insufficiency and anemia are at high risk of CIN. Baseline eGFR and baseline hemoglobin are independent predictors of CIN.

## Introduction

Contrast-induced nephropathy (CIN) is an iatrogenic disorder resulted from exposure to contrast media. The term CIN indicates an impairment of renal function (the elevation of serum creatinine by ≥0.5 mg/dl or ≥25 %) occurring within 3 days following the intravascular administration of contrast media, not attributable to other causes [1–3]. CIN is associated with increased morbidity and mortality, particularly in high-risk patients who have undergone coronary angiography and/or percutaneous coronary interventions. Although many studies demonstrate that preexisting renal dysfunction, diabetes mellitus, older age and reduced left ventricular systolic function are the most important risk factors for CIN, the association between baseline hemoglobin and CIN after injection of contrast agents has not been completely clarified. We hypothesize that anemic patients would be an increased risk of developing CIN due to renal ischemia. In this study, we examined the effect of anemia on the rates of CIN in patients undergoing percutaneous coronary intervention.

## Objects and methods

### Study population

Between January 1, 2008, and October 31, 2009, a total of 1,026 patients who had undergone coronary intervention procedure were enrolled in this study. Among them 622 were men and 404 women; median age was 64 (32–81 years). This study was conducted in accordance with the declaration of Helsinki. This study was conducted with approval from the Ethics Committee of Affiliated Hospital of Xuzhou Medical College. Written informed consent was obtained from all participants. Patients with reduced renal function were hydrated with 0.9 % saline at 1 ml/kg/h for 12 h before and after catheterization. For emergency coronary interventional procedures, physiologic (0.9 %) saline was given intravenously at a rate of 1 ml/kg/h for 12 h after contrast exposure. In patients with left ventricular ejection fraction (LVEF) <40 % or overt heart failure, the hydration rate was reduced to 0.5 ml/kg/h. A nonionic, low-osmolality contrast agent was used almost exclusively in our laboratory. All selective patients provided written informed consent for PCI.

### Study protocols and definitions

Serum creatinine concentrations were measured before and within 48 h of administration of contrast agents in every patient, and further measurements were performed in all patients developing CIN. Data were entered in a database that contained demographic, clinical and angiographic data. Anemia was defined as hemoglobin (Hgb) <120 g/l in women and <130 g/l in men, according to the World Health Organization criteria [4].

Renal function was assessed by the estimated glomerular filtration rate (eGFR) using the MDRD formula for Chinese patients [5]: GFR (ml/min/1.73 m^2^) = 175 × Scr (mg/dl)^−1.154^ × age^−0.203^ × (0.79 if female). This equation gives a more accurate assessment of renal function than serum creatinine alone.

Renal function was categorized according to the stages set by the National Kidney Foundation, with ≥90 ml/min normal, 60–89 ml/min mildly impaired, 30–59 ml/min moderately impaired and <30 ml/min severely impaired.

Contrast-induced nephropathy (CIN) was defined as the elevation of serum creatinine by ≥0.5 mg/dl or ≥25 % occurring within 3 days after the intravascular administration of contrast medium, without an alternative etiology [1, 2].

### Statistical analysis

Continuous variables are expressed as mean ± standard deviation (SD), and categorical data were presented as absolute values and percentages. *t* test and one-way analysis of variance (ANOVA) with post-Sheffe-type comparison test were used for parametric comparison. Mann–Whitney *U* and Kruskal–Wallis test were used for nonparametric comparison. Chi-square or the Fisher’s exact tests were used for comparison of categorical variables as required. Multivariate predictors of CIN were identified by logistic regression using stepwise selection. A two-sided 95 % confidence interval (CI) was constructed around the point estimate of the odds ratio (OR). The variables chosen by the model included all the potential confounding variables. All hypothesis testing was two tailed. A *P* value <0.05 was considered as statistically significant. Analysis was performed by using SPSS 13.0 statistical software.

## Results

### Baseline clinical characteristics

The baseline clinical characteristics of patients with CIN and non-CIN are summarized in Table 1. Of the 1,026 patients in this study, diabetes mellitus was present in 388 (37.8 %) and anemia in 222 (21.6 %) of patients at baseline. Thirty-two patients (3.1 %) experienced CIN after the procedure. These patients were significantly older with a higher incidence of anemia.

**Table 1:** Baselines clinical characteristics

Characteristic	CIN (*n* = 32)	Non-CIN (*n* = 994)	*P* value
Age (years)^a^	66.5 ± 14.2	61.2 ± 13.6	0.03
Male gender	22 (68.8)	693 (69.7)	0.91
Body mass index (kg/m^2^)^a^	26 ± 4	25 ± 6	0.73
Hypertension	15 (46.9)	439 (44.2)	0.76
Hypercholesterolemia	11 (34.3)	354 (35.6)	0.89
Diabetes mellitus	12 (37.5)	376 (37.8)	0.97
LVEF	0.51 ± 0.16	0.52 ± 0.15	0.94
Anemia	14 (43.4)	208 (20.9)	0.002
AMI	6 (18.8)	191 (19.2)	0.94
UAP	14 (43.8)	426 (42.9)	0.92
Prior myocardial infarct	5 (15.6)	148 (14.7)	0.91

*LVEF* left ventricular ejection fraction, *AMI* acute myocardial infarction, *UAP* unstable angina pectoris

^a^Mean ± SD

### Laboratory data

Patients who developed CIN had a higher baseline serum creatinine and a lower eGFR (Table 2). In comparison with patients without CIN, patients with CIN also had higher blood glucose levels and more often presented anemia.

**Table 2:** Laboratory data in patients with and without CIN

Characteristics	CIN (*n* = 32)	Non-CIN (*n* = 994)	*P* value
Serum creatinine (mg/dl) ≥1.5	17 (53.1 %)	88 (8.9 %)	<0.01
Baseline	2.36 ± 2.36	1.12 ± 1.85	<0.01
After catheterization	3.38 ± 2.91	1.08 ± 0.78	<0.01
eGFR (ml/min/1.73 m^2^) <60	22 (68.8 %)	236 (23.7 %)	<0.01
Baseline	36 ± 23	89 ± 39	<0.01
After catheterization	32 ± 21	88 ± 37	<0.01
Glucose (mmol/l)	8.3 ± 4.7	7.4 ± 3.4	0.04
The amount of the contrast agent (ml)	182 ± 46	176 ± 48	NS
Hemoglobin (g/l)	126 ± 22	136 ± 19	<0.01

### Incidence of CIN in patients with anemia

**Fig. 1:**
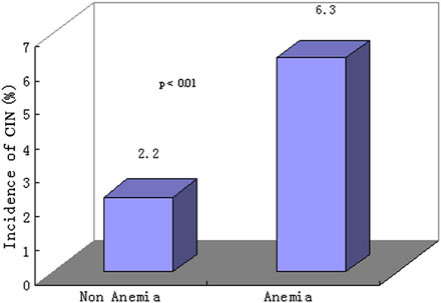
Incidence of CIN in anemic and non-anemic patients. The incidence of CIN in anemic patients was significantly higher than in non-anemic patients (6.3 vs. 2.2 %; *P* < 0.01)

As shown in Fig. 1, the incidence of CIN in anemic patients (hemoglobin <12 g/dl in women and <13 g/dl in men) was significantly higher than in non-anemic patients (6.3 vs. 2.2 %; *P* < 0.01).

**Fig. 2:**
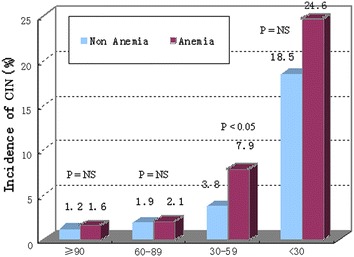
Incidence of CIN in patients with anemia and reduced baseline eGFR. The incidence of CIN increased with decreasing of baseline eGFR in both the anemia and non-anemia groups. In patients with baseline eGFR <30 ml/min, a high proportion of both anemic and non-anemic patients experienced CIN (24.6 vs. 18.5 %; *P* = NS). When baseline eGFR was 30–59 ml/min, the incidence of CIN in anemic patients was twofold higher than in non-anemic patients (7.9 vs. 3.8 %; *P* < 0.05). Among patients with baseline eGFR 60–89 ml/min and **≥**90 ml/min, there was no significant difference in the incidence of CIN between anemic and non-anemic patients (2.1 vs. 1.9 %, *P* = NS; 1.6 vs. 1.2 %; *P* = NS)

The incidence of CIN increased with decreasing of baseline eGFR in both the anemia and non-anemia groups. In patients with baseline eGFR <30 ml/min, a high proportion of both anemic and non-anemic patients experienced CIN (24.6 vs. 18.5 %; *P* = NS, Fig. 2). When baseline eGFR was 30–59 ml/min, the incidence of CIN in anemic patients was twofold higher than in non-anemic patients (7.9 vs. 3.8 %; *P* < 0.05). Among patients with baseline eGFR 60–89 ml/min and ≥90 ml/min, there was no significant difference in the incidence of CIN between anemic and non-anemic patients (2.1 vs. 1.9 %, *P* = NS; 1.6 vs. 1.2 %; *P* = NS).

### Multivariate logistic regression analysis

Multivariate logistic regression analysis revealed baseline eGFR and hemoglobin as independent predictors of CIN after percutaneous coronary intervention. The variables included in the first step of these multivariate analysis were age, sex, BMI, hypertension, hypercholesterolemia, LVEF, presence of diabetes mellitus, AMI, UAP, prior MI, baseline eGFR, amount of contrast agent administered, glucose level and hemoglobin level. Anemia was also an independent predictor of CIN (OR 2.352, 95 % CI 1.395–3.453, *P* < 0.001) when it was introduced into the multivariate model instead of baseline hemoglobin.

## Discussion

Contrast-induced nephropathy is an important complication in the use of iodinated contrast media, which accounts for a significant number of cases of hospital-acquired CIN [1]. With increasing number of diagnostic and therapeutic catheterizations each year, particularly among patients who may have serious conditions predisposing to CIN, the incidence of CIN will continuously increase. The ability of effective prevention of CIN in high-risk patients will provide significant public health benefits as we potentially reduce the inhospital mortality rate, the length of hospital stay and the subsequent use of chronic hemodialysis.

In the present study, the incidence of CIN was 3.1 % in the consecutive patients undergoing percutaneous coronary intervention. Post-procedure creatinine concentration was measured within 48 h or before discharge. We may have missed a later increase in serum creatinine in some patients who did not have renal function deterioration within 48 h of their procedure. The incidence of CIN in our study was 3.1 %, which is slightly lower than the results of Rihal et al. [5]. In patients with severe renal insufficiency (baseline eGFR <30 ml/min), the incidence of CIN was 21.3 %. This was consistent with the previous studies, which suggested a higher incidence of CIN in patients with greater reduction in renal function [6–11]. In a series of 7586 patients undergoing cardiac catheterization, Rihal et al. [6] found a low risk (2.4 %) of CIN (defined as an increase in serum creatinine levels ≥0.5 mg/dl) in patients with normal renal function, but a high risk (30.6 %) in those with serum creatinine levels ≥3.0 mg/dl. Moore et al. [7] demonstrated a high, significant relationship between an increasing baseline level of serum creatinine and the frequency of nephrotoxicity (varying from 2 % in those with baseline creatinine of <1.5 mg/dl to 20 % in those with levels of >2.5 mg/dl). Recently, Liu et al. [12] found that the ratio of volume of contrast media to eGFR ≥2.39 was a significant and independent predictor of CIN after primary PCI in patients with STEMI. Rosenstock et al. [13] reported that patients with chronic kidney disease without evidence of CHF who receive adequate hydration appear to have a very low risk of CIN associated with angiography and a low EF (less than 40 %) appeared to be the most significant risk factors for CIN. The inhospital mortality rate in patients developing renal insufficiency is directly related to the magnitude of the increase in the serum creatinine concentration [14, 15]. Even small increments in serum creatinine can develop significant increase in morbidity and mortality [16]. Renal failure after contrast administration requiring inhospital dialysis is associated with poor outcome including 36 % inhospital mortality and 19 % 2-year survival [15].

It is well known that patients with a GFR <60 ml/min per 1.73 m^2^ are more likely to have anemia and that prevalence and severity of anemia increase with declining renal function [17]. Our study demonstrated that baseline hemoglobin was an independent risk factor for CIN in all patients. When anemia was introduced into the multivariate model instead of baseline hemoglobin, it was also an independent predictor of CIN. This finding paralleled the recent clinical trial finding of Nikolsky et al. [18], who found that lower baseline hematocrit was an independent predictor of contrast-induced nephropathy; each 3 % decrease in baseline hematocrit resulted in significant increase in the odds of contrast-induced nephropathy in patients with and without chronic kidney disease. Among 7,230 consecutive patients after percutaneous coronary interventions, Dangas et al. [19] showed that decreased eGFRs and lower baseline hematocrit were most significant independent predictors of CIN in patients with chronic kidney disease. What is the possible mechanism to explain that baseline hemoglobin is an independent predictor for CIN? In the pathophysiology of CIN, one main factor is a reduction in renal perfusion caused by a direct effect of contrast media on kidney. The outer medullary region is particularly susceptible to ischemic injury because of its high metabolic activity and low prevailing oxygen tension [20]. The partial oxygen pressure of the outer medulla in the kidney is very low during normal function. Contrast media aggravates hypoxic injury to this region by increasing renal vascular resistance. Kim et al. [21] reported that contrast media could increase oxygen affinity of hemoglobin, so oxygen delivery to the peripheral tissues might be impaired. Local renal hypoxia can be more aggravated in patients with low hemoglobin after exposure to contrast media; hence, the combination of contrast-induced vasoconstriction and anemia may decrease oxygen delivery sufficiently to cause renal medullary hypoxia. Thus, it is intuitive that anemia may play a role in CIN risk. Nikolsky et al. [18] demonstrated that patients with the lowest eGFR and hematocrit had the highest rates of CIN. In the present study, anemia is an independent risk factor for CIN in all patients. Anemia significantly increases the incidence of CIN in patients with moderate renal dysfunction. Patients with both preexisting renal insufficiency and anemia are at the highest risk of developing CIN. Before cardiac catheterization, correction of anemia especially in patients with preexisting renal failure might be a modifiable risk factor for CIN, even though this has to be proven by prospective randomized trials.

There were several study limitations in this study. First, the follow-up assessment of renal function in our study was 1–3 days after PCI; therefore, we might have missed a later increase in serum creatinine in some patients who did not have renal function deterioration within 48 h of their procedure. This might result in a slight underestimation of CIN. Second, we did not have the etiology of anemia in vast majority of anemic patients. In addition, we did not have data on erythropoietin levels and plasma volume information that might have provided better understanding of the role of low baseline hemoglobin in the development of CIN.

In conclusion, our study demonstrates that the overall incidence of CIN after PCI exposure in entire populations is low (3.1 %) using guideline-based recommendations for prophylaxis of CIN. Patients with both preexisting renal insufficiency and anemia are at high risk of CIN. Anemia is associated with increases in the incidence of CIN in patients with moderate renal dysfunction. Baseline eGFR and baseline hemoglobin (or anemia) are independent predictors of CIN.
